# Permanent Improved High-Quality Draft Genome Sequence of *Nocardia casuarinae* Strain BMG51109, an Endophyte of Actinorhizal Root Nodules of *Casuarina glauca*

**DOI:** 10.1128/genomeA.00799-16

**Published:** 2016-08-04

**Authors:** Faten Ghodhbane-Gtari, Nicholas Beauchemin, Moussa Louati, Imen Nouioui, Amir Ktari, Karima Hezbri, Abdellatif Gueddou, Amy Chen, Marcel Huntemann, Natalia Ivanova, Nikos Kyrpides, Victor Markowitz, Kostas Mavrommatis, Ioanna Pagani, Arnab Sen, Luis Wall, Tanja Woyke, Maher Gtari, Louis S. Tisa

**Affiliations:** a University of Tunis-El Manar, Tunis, Tunisia; b University of New Hampshire, Durham, New Hampshire, USA; c DOE Joint Genome Institute, Walnut Creek, California, USA; d University of North Bengal, Siliguri, India; e University of Quilmes, Quilmes, Argentina

## Abstract

Here, we report the first genome sequence of a *Nocardia* plant endophyte*, N. casuarinae* strain BMG51109, isolated from *Casuarina glauca* root nodules. The improved high-quality draft genome sequence contains 8,787,999 bp with a 68.90% GC content and 7,307 predicted protein-coding genes.

## GENOME ANNOUNCEMENT

Members of the genus *Nocardia* are ubiquitous soil-borne aerobic microorganisms belonging to a large group of actinobacteria that are best known for their pathogenicity and their harmful infections for humans or animals (1). However, several *Nocardia* spp. have been consistently reported as plant endophytes and isolated from disinfected roots (*N. callitridis* [2]), stems (*N. artemisiae* [3] and *N. endophytica* [4]), and actinorhizal root nodules (*N. autotrophica* [5], *N. casuarinae* [6], and *Nocardia* sp. [7–9]). The potential role of the *Nocardia* endophyte has been postulated, especially in the case of actinorhizal symbiosis (5, 10). To the best of our knowledge, these plant-associated *Nocardia* spp. have not had their genomes sequenced.

We report here the genome sequence of *Nocardia casuarinae* strain BMG51109, which was isolated from root nodules of *Casuarina glauca* growing in Tunisia (6). *N. casuarinae* strain BMG51109 was sequenced to provide new insights into the role that this microbe plays in actinorhizal plants.

The draft genome of *N. casuarinae* strain BMG51109 was generated at the DOE Joint Genome Institute (JGI; Walnut Creek, CA, USA) using Pacific Biosciences (PacBio) technology. A PacBio SMRTbell library was constructed and sequenced on the PacBio RS platform, which generated 583,909 filtered subreads totaling 1.4 Gbp. All general aspects of library construction and sequencing performed at the JGI can be found at http://www.jgi.doe.gov. The raw reads were assembled using HGAP version 2.0.0 (11). The final draft assembly contained 4 contigs in 4 scaffolds, totaling 8.8 Mbp in size. The input read coverage was 155.7×.

Project information is accessible in the Genomes Online Database (12). Genes were identified using Prodigal (13), followed by a round of manual curation using GenePRIMP (14) as part of the microbial annotation pipeline of the JGI (15). Additional gene prediction analysis and manual functional annotation were performed within the Integrated Microbial Genomes–Expert Review (IMG-ER) platform (http://img.jgi.doe.gov) developed by JGI (16).

The high-quality draft genome of *N. casuarinae* strain BMG51109 was resolved to 4 scaffolds consisting of 7,730,817 bp, with a G+C content of 68.90%, 7,822 candidate protein-coding genes, 55 tRNA genes, and 3 rRNA regions.

### Nucleotide sequence accession numbers. 

The *Nocardia casuarinae* strain BMG51109 genome sequence has been deposited at DDBJ/EMBL/GenBank under the accession number JAFQ00000000. The version described in this paper is the first version, JAFQ01000000.
